# ZBP1 and IFI16: novel innate sensors and inflammation regulation under infection stress

**DOI:** 10.3389/fimmu.2026.1869593

**Published:** 2026-09-22

**Authors:** Chengbin Lu, Benmo Xu, Yu Xiong

**Affiliations:** 1 The Second Xiangya Hospital, Central South University, Changsha, Hunan, China; 2 The First Affiliated Hospital of Kunming Medical University, Kunming, Yunnan, China

**Keywords:** ZBP1, IFI16, innate immunity, nucleic acid sensor, PANoptosis, pyroptosis, inflammation regulation, infection

## Abstract

The innate immune system is the first line of defense against infection, using pattern recognition receptors to sense pathogens and abnormal host-derived nucleic acids. Among these, Z-DNA–binding protein 1 (ZBP1) and interferon gamma–inducible protein 16 (IFI16) are two important nuclear and cytosolic nucleic acid sensors attracting recent attention. These sensors play key roles in detecting abnormal nucleic acids from viruses, bacteria, and damaged host cells. They trigger downstream innate immune responses to regulate inflammation. Despite much progress, key questions remain about how they are exactly activated under infection stress, the complexity of their signaling networks, and their dual roles in protective immunity and pathological inflammation. This review summarizes the structural features of ZBP1 and IFI16, how they recognize ligands, and their interactions with key signaling molecules (including RIPK1, RIPK3, MLKL, and caspase-1). We then emphasize cell fate decisions mediated by these sensors. Especially, ZBP1 is the core upstream regulator of PANoptosis (an integrated form of programmed cell death combining pyroptosis, apoptosis, and necroptosis via the PANoptosome complex), alongside classical cell death pathways. In addition, we review recent advances to highlight functional roles of ZBP1 and IFI16 in herpesvirus, influenza virus, and bacterial infections, and their dysregulation in autoimmune diseases. For IFI16, human studies and murine homolog-based evidence are analyzed. Further we review unique and overlapping functions, including a comparison with other canonical nucleic acid sensors (e.g., cGAS, AIM2, RIG-I) and analyze the ADAR1-ZBP1 axis in self-nonself nucleic acid discrimination. We cover the therapeutic potential of targeting ZBP1- and IFI16-mediated pathways for anti-inflammatory and anti-infective strategies and provide a balanced analysis of developmental challenges, potential toxicity, and risks of impaired immune surveillance.

## Introduction

The innate immune system is the first line of host defense against invading pathogens. It relies on pattern recognition receptors (PRRs) to detect pathogen-associated molecular patterns and damage-associated molecular patterns (1–3). Z-DNA–binding protein 1 (ZBP1) and interferon gamma–inducible protein 16 (IFI16) are two interferon-inducible nucleic acid sensors. They attracted attention because they can recognize different types of abnormal nucleic acids in both the nucleus and the cytosol. Thus, they start innate immune responses and regulate the process of inflammation (4–6). ZBP1 detects left-handed Z-form nucleic acids (Z-DNA/Z-RNA) produced during viral infection and cellular stress, which will trigger programmed cell death and inflammation. By contrast, IFI16 senses double-stranded DNA in nuclear and cytosolic compartments to drive type I interferon responses and inflammasome activation (7, 8).

Despite much progress in the field, there are still key questions about how ZBP1 and IFI16 are exactly activated under infectious stress, how complex their signaling networks are, and how their dysregulation causes pathological inflammation. This review studies ZBP1 and IFI16 side by side, focusing on three interconnected themes. Both sensors are interferon-inducible and work beyond canonical membrane-bound PRRs, surveying nuclear and cytosolic compartments. Their abnormal activation is linked to severe infectious and autoimmune conditions (including influenza, herpesvirus infection, systemic lupus erythematosus, and ulcerative colitis) (9–11). They come together on shared inflammatory and cell death pathways, like PANoptosis, pyroptosis, necroptosis, and type I interferon signaling. However they fundamentally differ in ligand specificity, subcellular localization, and signaling dynamics (12–14). The ADAR1–ZBP1 regulatory axis is also discussed as a checkpoint for the discrimination of self-nonself. We also review post-translational modifications that adjust sensor activity, and the therapeutic potential and risks of targeting these pathways in infectious, autoimmune, and malignant diseases.

## Molecular structures and recognition mechanisms of ZBP1 and IFI16

### Structural features of ZBP1 and recognition of Z-DNA/RNA

ZBP1 is a key nuclear and cytosolic nucleic acid sensor with a unique structural organization that enables the specific recognition of noncanonical left-handed nucleic acids, namely Z-DNA and Z-RNA (15–17). The N-terminus of ZBP1 contains two tandem Zα domains (Zα1 and Zα2), which constitute the core modules responsible for sensing Z-form nucleic acids (24). Biochemical and functional studies indicate that Zα2 is the primary functional domain required for ligand binding and downstream signaling activation. Whereas Zα1 retains Z-DNA/RNA binding capacity, its non-redundant functional role in initiating downstream signaling cascades remains incompletely defined, with limited *in vivo* experimental evidence in support (18, 19). These Zα domains bind Z-DNA and Z-RNA with high affinity, and this interaction represents a critical initiating step for downstream immune signaling and cell death programs (19). During viral infection, viruses such as influenza A virus (IAV) and poxviruses generate Z-RNA–containing intermediates during replication or transcription. These structures are specifically detected by the Zα domains of ZBP1, thereby triggering host antiviral defense responses (20). Within the nucleus, ZBP1 can also recognize Z-form nucleic acids generated during active viral transcription and trigger nuclear lytic cell death, providing an early line of defense prior to cytosolic viral egress (20).

Instead, the functional necessity of the second RIP Homotypic Interaction Motif (RHIM2) remains less definitively validated by loss-of-function studies. Recent structural and mutagenesis analyses indicate that RHIM2 may contribute to the stabilization of the higher-order ZBP1–RIPK3 complex and facilitate the recruitment of additional RHIM-containing proteins, such as RIPK1 under specific condition (21, 22). RHIM2 has been proposed to play auxiliary or context-dependent roles—for example, enhancing signal amplification when RHIM1-mediated interactions are suboptimal or when Z-nucleic acid ligand abundance is low—rather than serving as an obligate signaling module (17, 21, 22). RHIM2-specific knockout or point-mutation models in physiologically relevant infection settings will be needed to resolve its precise functional contribution.

### Structural features of IFI16 and DNA recognition

IFI16, a member of the Pyrin and HIN domain-containing protein family (PYHIN protein family), functions as an important predominantly nuclear DNA sensor with active cytosolic signaling roles via regulated nucleocytoplasmic shuttling. Structurally, IFI16 is characterized by an N-terminal pyrin domain (PYD) and two C-terminal HIN domains (HIN-A and HIN-B) (11). The HIN domains mediate sequence-independent binding to double-stranded DNA (dsDNA), allowing IFI16 to detect both invading viral DNA and aberrant host-derived DNA in the nuclear and the cytosolic compartments (4, 23).

Functionally, IFI16 can act both as an upstream sensor and as a scaffold to enhance cyclic GMP-AMP synthase (cGAS)–STING signaling. Structural and biochemical studies show that IFI16 directly interacts with cGAS via its HIN domains, promoting cGAS binding to DNA and enhancing 2′, 3′-cyclic GMP-AMP (cGAMP) synthesis, while its pyrin domain (PYD) engages STING to facilitate TBK1–IRF3 recruitment (10, 24, 25). IFI16 has also been reported to interact with STING independently of cGAS in certain contexts, suggesting a cGAS-independent IFI16–STING signaling axis may operate under specific conditions (26, 27). This multilayered crosstalk between the two DNA sensing systems is thought to be important for effective antiviral and antibacterial immunity.

IFI16 recognition of DNA is closely linked to its ability to oligomerize. Multiple IFI16 molecules assemble along the recognized DNA to form higher-order complexes, thereby amplifying downstream signaling (11). The N-terminal PYD mediates interactions with downstream signaling molecules. Through PYD, IFI16 can engage STING to activate the TANK-binding kinase 1 (TBK1)–interferon regulatory factor 3 (IRF3) signaling axis, leading to the production of type I interferons. Alternatively, IFI16 can interact with apoptosis-associated speck-like protein containing a CARD (ASC), promoting the formation of ASC specks, activation of caspase-1, inflammasome assembly, and subsequent maturation of interleukin-1β (IL-1β) (26, 28).

IFI16 has been shown to sense uncoated viral genomes or damaged host DNA, playing a critical role in antiviral immunity as well as in autoinflammatory diseases (29). Within the nucleus, IFI16 directly detects herpesviral genomes during early infection prior to their egress to the cytosol, triggering both interferon responses and viral genome silencing. Upon activation, IFI16 undergoes nucleocytoplasmic translocation to propagate signaling to cytosolic STING and inflammasome components, bridging nuclear sensing to cytosolic effector pathways. Notably, IFI16 expression is significantly elevated in peripheral blood mononuclear cells from patients with systemic lupus erythematosus (SLE) and correlates closely with disease activity, suggesting its potential utility as a biomarker (29). It is important to note that IFI16 is primarily present in primates and lacks a direct ortholog in mice; consequently, mechanistic studies are largely conducted in human-derived cell systems. The murine PYHIN family member p204 (encoded by Ifi204) serves as the closest functional analog, sharing similar domain architecture and DNA-sensing capacity but with non-identical regulatory and signaling properties (11, 19). Throughout this review, findings from human IFI16 studies and murine p204 studies are explicitly distinguished to avoid cross-species extrapolation artifacts. The distinct structural domains of ZBP1 and IFI16 and their respective downstream signaling pathways are illustrated in **Figure 1**.

**Figure 1:**
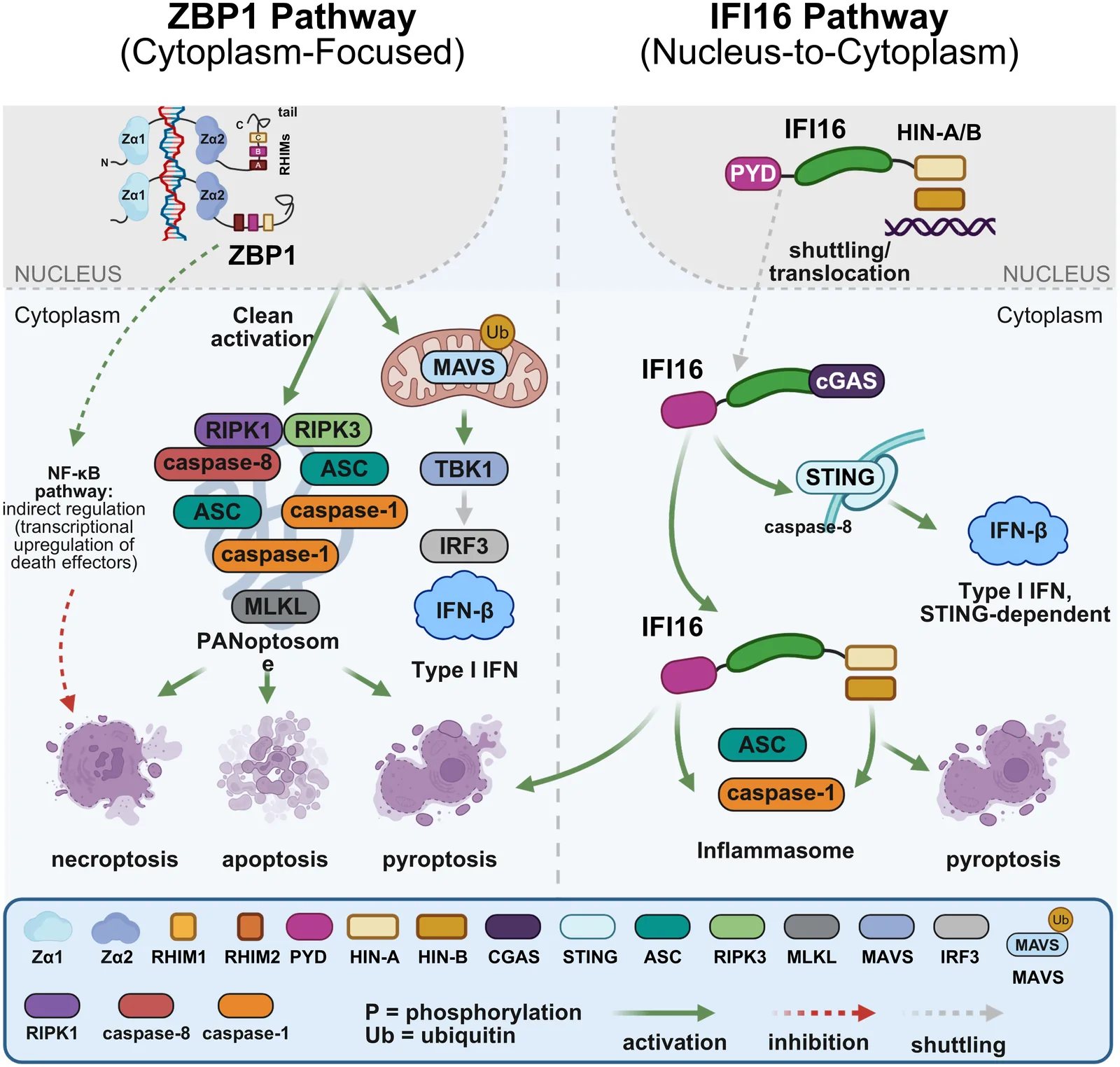
Overview of the molecular architecture and signaling networks of ZBP1 and IFI16. The left panel illustrates the Zα and RHIM domains of ZBP1, its recognition of Z-form nucleic acids (Z-RNA/Z-DNA) in both nuclear and cytosolic compartments, interaction with RIPK1/3, and activation of PANoptosis, necroptotic, and apoptotic signaling pathways, as well as NF-κB and type I interferon responses (11, 19). The right panel depicts the PYD and HIN domains of IFI16, its nucleocytoplasmic shuttling, nuclear sensing of chromatinized viral DNA, cytosolic signaling through the STING–IRF3 axis (type I interferon response) and the ASC–caspase-1 pathway (pyroptosis/IL-1β maturation), and its cooperative interplay with cGAS (10, 24, 25). The NF-κB link to cell death is depicted as indirect (dashed arrow) to reflect that NF-κB primarily drives inflammatory transcription rather than directly executing necroptosis, with contextual crosstalk occurring via transcriptional upregulation of cell death machinery components. Created in BioRender. Lu, C. (2026) (https://BioRender.com/7s75aco).

## Activation and downstream signaling pathways under the stress of infection

### ZBP1-mediated signaling: from the responses of interferon to programmed cell death

ZBP1 can start several downstream signaling pathways when it recognizes Z-form nucleic acids (Z-RNA/Z-DNA) produced during pathogen infection or cellular stress. To limit the replication of pathogens, ZBP1 induces programmed cell death (21, 30). One recent advance in this field comes from the evidence that ZBP1 acts as a core upstream sensor and scaffold for the PANoptosome, a multiprotein complex driving PANoptosis (20, 30). PANoptosis is an integrated form of regulated cell death that simultaneously exhibits features of pyroptosis, apoptosis, and necroptosis (30, 31). After binding with Z-nucleic acid, ZBP1 recruits RIPK3 in a RHIM-dependent way to nucleate the PANoptosome complex, and then RIPK3 brings in caspase-8, ASC, caspase-1 and MLKL to turn on all three cell death axes together. In addition, this integrated death program is also useful to efficiently eliminate infected cells even if pathogen-encoded evasion factors block individual cell death pathways. In viral infection models, the laboratories of Kanneganti and Balachandran pointed out this mechanism in detail (20, 30–32). Through its N-terminal Zα domains, ZBP1 binds Z-form nucleic acids specifically. After that, it connects with receptor-interacting serine/threonine-protein kinases RIPK1 and RIPK3 using its C-terminal RHIM domains, so a necroptotic signaling complex is built (33). In this complex, RIPK3 gets activated to phosphorylate mixed lineage kinase domain-like protein (MLKL), and then MLKL moves to the plasma membrane to make pores, which will lead to necroptosis at the end (13). This is one core antiviral defense mechanism. In the example of influenza A virus infection, ZBP1 senses virus-derived Z-RNA and starts RIPK3-dependent necroptosis, so that the infected cells are cleared (33). When necroptosis is blocked, for example in the absence of RIPK3 or MLKL, the ZBP1-RIPK3 complex can recruit and turn on caspase-8. This starts the extrinsic apoptotic pathway, providing an alternative mechanism for pathogen clearance (34–36). This signaling switch ensures that infected cells can still be killed when necroptosis is stopped, showing redundancy and fine-tuned regulation of host defenses.

Through RHIM-dependent interactions with RIPK1 and RIPK3, ZBP1 also drives the activation of nuclear factor κ-light-chain-enhancer of activated B cells (NF-κB) (21, 30). For the mechanism part, the linear ubiquitin chain assembly complex (LUBAC) and TAK1 kinase are recruited by the ZBP1-RIPK3 complex, and this causes inhibitor of NF-κB (IκB) phosphorylation and NF-κB nuclear translocation. This pathway mainly helps to transcribe proinflammatory cytokines and chemokines and does not execute cell death directly. The crosstalk between NF-κB and necroptosis is indirect, given that NF-κB upregulates the expression of cell death components (e.g., MLKL, RIPK3) and inflammatory mediators that augment sensitivity to necroptotic signaling, instead of phosphorylating necroptotic mediators directly. The NF-κB-necroptosis link is shown in **Figure 1** as a dashed arrow, to point to this indirect and context-dependent crosstalk (21, 30).

Besides, some studies demonstrated that ZBP1 can activate the TBK1-IRF3 signaling axis. Nassour et al. (2023) provided the first evidence that ZBP1 mediates signals through mitochondrial antiviral signaling protein (MAVS) to induce type I interferons (e.g., IFN-β) when a replicative crisis happens (37). ZBP1 localizes to dysfunctional telomeres and links with the MAVS adaptor on mitochondria. This triggers TBK1-IRF3 phosphorylation and produces interferons without relying on classical RNA sensors (37, 38). However, increasing evidence shows that under infectious stress, ZBP1 predominantly drives cell death rather than interferon production. In the woodchuck hepatitis virus (WHV) infection model, the activation of ZBP1 suppresses viral replication and induces cell death, without relying heavily on interferon signaling (39). This defense strategy focuses on cell death, and it can clear viral replication niches very fast to limit the spread of virus more effectively.

### IFI16-mediated signaling: complementation and independence of cGAS-STING axis

IFI16 works as one important DNA sensor in the nucleus and cytosol. When it detects exogenous or endogenous DNA, it starts the innate immune response in many ways. It acts as a complement to cGAS-STING pathway and it is also an independent signaling mediator (40). As a scaffold protein, IFI16 facilitates the activation of the cGAS-STING pathway (10, 24). After binding cytosolic DNA, IFI16 interacts with cyclic GMP-AMP synthase (cGAS). Consequently, cGAS recognizes DNA more efficiently and synthesizes cyclic GMP-AMP (cGAMP), and after that, STING is activated more efficiently to finally induce type I interferons and proinflammatory cytokines (25, 27). This amplifies interferon responses triggered by DNA, necessary for effective antiviral immunity. However, IFI16 can work independently of cGAS. Past studies showed that IFI16 uses its pyrin domain to interact with STING directly, so it skips cGAS to activate STING-TBK1-IRF3 signaling axis (26, 41, 42). For some specific cell types, IFI16 can even pull TBK1 and IRF3 directly together to build signaling complexes, to promote transcription of interferon genes (43). To sum up, the existence of these alternative pathways gives a backup mechanism for DNA sensing and signaling, especially meaningful when cGAS loses its function or is not present.

Parallel with interferon signaling, IFI16 activates NF-κB through two pathways: STING-dependent and STING-independent. STING recruited by IFI16 connects with the IκB kinase (IKK) complex, so that NF-κB nuclear translocation and transcription of proinflammatory cytokine genes are activated. In addition, IFI16-mediated inflammasome activation promotes the release of IL-1 family cytokines. These cytokines will further amplify NF-κB activation in bystander cells through paracrine signaling, eliciting a feedforward inflammatory loop (26, 44).

Beyond inducing interferon, IFI16 is critical for inflammasome activation and pyroptotic cell death. IFI16 interacts with apoptosis-associated speck-like protein containing a CARD (ASC) by using its pyrin domain. This forms canonical inflammasome complexes that bind and activate caspase-1. Because of this, caspase-1 cuts pro-IL-1β and pro-IL-18 to mature them into secreted forms, and this triggers strong inflammatory responses. At the same time, caspase-1 cleaves gasdermin D (GSDMD) to release its N-terminal fragment. This fragment forms membrane pores and leads to pyroptosis. This process eliminates infected cells and amplifies the immune responses via proinflammatory mediators (44, 45). Further, IFI16 helps host defense against RNA viruses like influenza A virus, extending beyond classical DNA sensing. As Jiang et al. (2021) pointed out in Nature Microbiology, IFI16 directly senses viral RNA and improves RIG-I transcription and activation during influenza infection to limit viral replication (6). In respiratory epithelial cells, IFI16 also regulates the inflammatory signaling and cell death pathways caused by the virus. This shows one extra layer of host protection against RNA viruses (6). IFI16-mediated inflammasome activation is involved in many other diseases. For example, in the salivary glands of patients with primary Sjögren’s syndrome (pSS), the expression of IFI16 increases. This correlates with expression of pyroptosis-related genes and suggests that IFI16 takes part in the disease-associated inflammation (46). In a similar way, patients with active ulcerative colitis (UC) have markedly increased IFI16 expression in their colonic mucosa. This is connected with the activation of DNA-sensing pathways and type I interferon signaling, supporting a critical role for IFI16 in intestinal inflammation (47).

## Functional roles in specific pathogen infection models

### Central roles in antiviral defense: the examples of herpesviruses and orthomyxoviruses

During herpesvirus infection, human IFI16 primarily detects viral DNA in the nucleus. Before relocation to the cytosol, it recognizes both naked and chromatinized herpesviral genomes from as early as entering the nucleus and starts the responses of interferon and viral genome silencing (48). Some studies in murine cells support that the functional homolog p204 shows similar DNA-sensing ability during murine cytomegalovirus (MCMV) infection, although species-specific differences may still exist (24). In one woodchuck model of chronic hepatitis B virus (HBV) infection, the HSV-60 agonist activates the IFI16 pathway and greatly stops the replication and secretion of WHV, suggesting that IFI16 may directly sense viral DNA and start antiviral effects (39). At the same time, ZBP1 is necessary for sensing orthomyxoviruses like influenza A virus. Through its Zα domains, ZBP1 detects left-handed Z-RNA generated during the replication of this virus to promote RIPK3-dependent necroptosis and PANoptosis. This process is key for the host ability to clear infected cells and stop viral dissemination. Mice without ZBP1 show higher sensitivity to influenza virus infection, which further highlights its essential role in antiviral immunity (14, 49). Besides the basic DNA-sensing role, IFI16 also helps anti-influenza immunity in human respiratory epithelial cells. IFI16 interacts with influenza viral RNA directly to promote RIG-I expression and its antiviral signaling to limit viral replication. Furthermore, IFI16 regulates the production of inflammatory cytokine and cell death pathways during influenza infection, and this will shape the severity of pulmonary immunopathology (6). Some new evidence also shows possible crosstalk and cooperation between ZBP1 and IFI16. However, direct physical interaction between ZBP1 and IFI16 has not been clearly demonstrated. These sensors share downstream signaling adaptors, including STING, RIPK3, and ASC, and common effector pathways (like NF-κB, type I interferons, and inflammasome activation). In woodchuck model of acute hepatitis B, we can see the significant upregulation of multiple pattern recognition receptors in liver, IFI16 and ZBP1. This change is closely related to generating virus-specific B-cell and T-cell responses and clearing the virus at the end (50–52). Thus, beyond restricting viral replication, ZBP1 and IFI16 also promote the activation of adaptive immunity and the establishment of long-term immune protection in the host. In MCMV and other complex infection models, coordinated activation of both sensors may help adjust the size and speed of immune responses. However further mechanistic studies are needed to reveal the molecular basis of this cooperation (7, 8).

### The roles in bacterial infection and host damage responses

Besides their antiviral functions, ZBP1 and IFI16 also take important parts in bacterial infections and host damage responses. For intracellular bacterial pathogens like Listeria monocytogenes and Salmonella spp., the host cells make use of multiple pattern recognition receptors to sense bacterial DNA (14). As a member of PYHIN protein family, human IFI16 and its murine functional homolog p204 can sense bacterial DNA released into the cytosol, and they can also sense host-derived DNA leaking from damaged cells during infection. This sensing leads to inflammasome activation, with consequent maturation and secretion of IL-1β and IL-18 and induction of pyroptosis. These processes are critical to clear intracellular bacteria (14, 53). In severe systemic inflammation, like during sepsis, host cells under stress or injury release endogenous DNA molecules, including mitochondrial DNA. These endogenous nucleic acids can be recognised by sensors such as ZBP1 and IFI16. Note that ZBP1 can sense exogenous and endogenous Z-form nucleic acid structures (54–56). Under noninfectious stress conditions, like heatstroke, ZBP1 is shown to react to endogenous ligands and regulate cell death and inflammatory responses, which highlights its possible part in the pathogenesis of systemic inflammatory response syndrome (57, 58). This ability to detect damage-associated molecular patterns adds to the functional scope of ZBP1 and IFI16, beyond pathogen defense, potentially involved in tissue homeostasis and pathological inflammation. **Figure 2** summarizes the division of labor between these two sensors in viral and bacterial infections.

**Figure 2:**
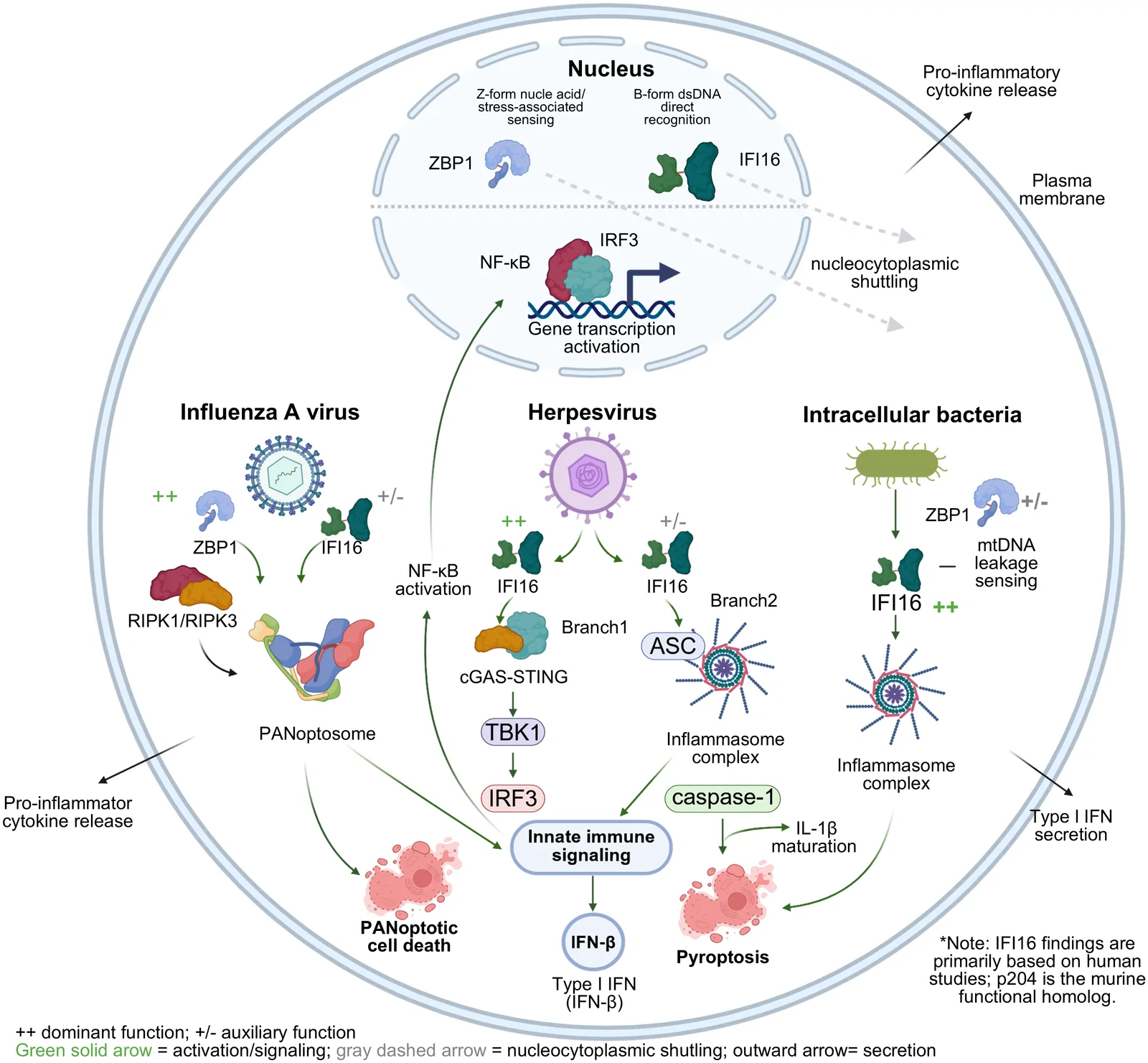
Functional divergence of ZBP1 and IFI16 in response to distinct pathogen infections. This figure highlights the differential sensing principles and functional roles of ZBP1 and IFI16 across infectious contexts. IFI16 directly recognizes B-form dsDNA from herpesviruses and intracellular bacteria, driving type I interferon responses and pyroptosis. ZBP1 senses Z-form nucleic acids generated during influenza A virus infection and cellular stress, predominantly driving necroptosis and PANoptosis. Both sensors converge on shared inflammatory effector pathways (NF-κB, proinflammatory cytokines) to coordinate host defense. This convergence is inferred from shared downstream components rather than demonstrated direct physical interaction. It should be noted that IFI16 findings are primarily derived from human systems; murine studies use the functional homolog p204. IAV, Influenza A Virus; HSV, Herpes Simplex Virus; ASC, Apoptosis-associated Speck-like Protein; IFN, Interferon; IL-1β, Interleukin-1 beta; cGAS, Cyclic GMP-AMP Synthase; STING, Stimulator ofInterferon Genes. Created in BioRender. Lu, C. (2026) (https://BioRender.com/w4zou57).

### The ADAR1-ZBP1 axis: one key checkpoint for self-nonself nucleic acid discrimination

Adenosine Deaminase Acting on RNA 1 (ADAR1) has two major isoforms with different subcellular distribution and regulatory functions. These are the constitutively expressed nuclear p110 isoform and the interferon-inducible cytoplasmic p150 isoform. The p150 isoform has one N-terminal Z-DNA/Z-RNA binding domain (Zα) to target it to Z-RNA substrates. Because of its cytoplasmic localization, it can directly compete with ZBP1 for endogenous Z-RNA binding. Therefore, it acts as the main inhibitor of ZBP1 activation in the cytosol (34). Conversely, the nuclear p110 isoform edits self-derived Alu repeat-containing RNA duplexes in nucleus, which prevents them from moving to cytoplasm (34, 36). Otherwise, these might adopt Z-conformations and activate ZBP1 there (34, 35). From a mechanistic perspective, the Zα domain of ADAR1 p150 can recognize and bind Z-RNA with high affinity, positioning the catalytic deaminase domain to trigger A-to-I editing for adjacent adenosines. Through this editing, I·U wobble base pairs are added in (34, 36). Because these pairs thermodynamically go against Z-helix formation, the structure of RNA will be reversed back to A-form (34, 36). In the intestine, unbalances within the ADAR1-ZBP1 axis drive epithelial cell death and chronic inflammation. When intestinal epithelial cells lose ADAR1 expression, this leads to ZBP1-dependent necroptosis and barrier dysfunction (34, 59). In addition to the editing activity, ADAR1 has editing-independent inhibitory effects on ZBP1 by direct protein-protein interaction. The Zα domain of ADAR1 p150 competitively inhibit the Zα domains of ZBP1. In this way, ZBP1 oligomerization and RHIM-dependent signalosome assembly are blocked sterically (34, 35). Hence, enzymatic RNA editing and direct steric blockade constitute a dual inhibitory mechanism ensuring strong suppression for ZBP1 under physiological conditions. The disruption of ADAR1-ZBP1 axis is widely recognized as one pathogenic driver in Aicardi-Goutières syndrome (21, 30). Disruption of this axis has also been implicated in other autoinflammatory and autoimmune disorders (21, 30).

Involvement in Inflammatory and Immune Disorders

The activation of ZBP1 and IFI16 is critical for pathogen recognition and response. However, excessive activation of ZBP1 and IFI16 exacerbates cell death and cytokine release, which triggers tissue damage and an inflammatory cytokine storm systemically (7, 14). The abnormalities of these pathways show a positive correlation with the severity of severe influenza, COVID-19, and some autoinflammatory disorders (7, 14). For patients with active UC, the expression of DNA sensor genes like IFI16 and ZBP1 increases favoring type I interferon signaling pathway, and this over-activation can cause JAK-dependent inflammatory cell death in colonic organoids (47). Wang et al. (2020) pointed out in murine models that the necroptosis and PANoptosis of intestinal stem cells mediated by ZBP1 represent a key driver of bowel inflammation and epithelial barrier damage, which is triggered by genome instability and endogenous Z-RNA accumulation (59). When epithelial cells die under this condition, luminal antigens are released and mucosal immune cells are activated which prolongs chronic intestinal inflammation (59). In addition, the impaired editing activity of ADAR1 in colonic epithelium makes cells more sensitive to ZBP1-driven death, which worsens IBD progression (59). ZBP1 can trigger RIPK3-dependent necroptosis and inflammatory responses, resulting in severe pathological conditions like perinatal lethality, skin inflammation, and colitis in mouse models with RIPK1 or CASP8 deficiency (21, 34, 60). Under homeostatic conditions, cells use different negative feedback mechanisms to control the over-activation of ZBP1, such as the autoinhibition of RHIM domain, protein phosphorylation, and degradation through the ubiquitin-proteasome pathway (21, 29). Usually, this autoinhibition is only removed under specific conditions like infection or stress, so that downstream signaling cascades can be activated (29). Similarly, abnormal overexpression of IFI16 has a close relation with disease activity in systemic lupus erythematosus (SLE), contributing to the pathogenesis of the disease by mediating excessive production of IFN-β (29). To keep immune homeostasis, cells continue to evolve complex regulatory mechanisms to limit the activity of ZBP1 and IFI16 through ubiquitination, autophagy-mediated degradation, and endogenous inhibitory molecules (11, 29). In patients with primary Sjögren’s syndrome (pSS), monocytes often show an exaggerated response to the stimulation of STING pathway (61). However, the upregulation of some positive regulatory factors (like TRIM38 and TRIM56) may indicate pathways to control excessive inflammatory responses through negative feedback mechanisms (61). Imbalances in these regulatory pathways may be the very key reasons for the onset and progression of inflammatory cytokine storms and autoimmune diseases. The abnormal expression and pathological significance of ZBP1 and IFI16 in autoimmune and inflammatory disorders are summarized in **Table 1**.

**Table 1:** Aberrant expression and pathological significance of ZBP1 and IFI16 in autoimmune and inflammatory diseases.

Disease	Key sensor	Aberrant expression (sample source)	Major pathological mechanisms/downstream effects	Clinical/pathological significance	Representative references
Systemic lupus erythematosus (SLE)	IFI16	Significantly upregulated (peripheral blood mononuclear cells, PBMCs)	Hyperactivation of the STING–IRF3 pathway, leading to excessive production of type I interferons (IFN-α/β); promotes autoantibody (anti-dsDNA) generation	Positively correlated with disease activity (SLEDAI score) and anti-dsDNA antibody titers; potential biomarker for disease activity	Fu et al., Clin Chim Acta, 2021 (29)
Systemic lupus erythematosus (lupus nephritis)	ZBP1	Significantly upregulated (human lupus nephritis renal tissue, MRL/lpr mouse kidney)	Activates podocyte PANoptosis via the STAT2-ZBP1 axis, aggravating renal inflammation, glomerular injury and tubular damage	Positively correlated with renal pathological grade and proteinuria severity; ZBP1 inhibition significantly alleviates renal lesions in lupus nephritis mice	Wang et al., Phytomedicine, 2025 (62)
Ulcerative colitis (UC)	IFI16	Significantly upregulated (active colonic mucosa)	Activation of type I interferon signaling via the STING–TBK1–IRF3 axis; induction of JAK-dependent inflammatory cell death (pyroptosis/apoptosis)	Positively correlated with disease activity index (Mayo score) and inflammatory infiltration; JAK inhibition partially reverses IFI16-mediated epithelial injury	Flood et al., Am J Physiol Gastrointest Liver Physiol, 2022 (47)
Ulcerative colitis (UC)	ZBP1	Elevated expression (active colonic mucosa)	Sensing of endogenous Z-RNA/DNA, triggering RIPK1/RIPK3-dependent necroptosis and PANoptosis; promotes IL-1β and IL-18 release; drives intestinal stem cell death via genome instability	Can drive severe colitis under conditions of impaired RIPK1 or CASP8; may contribute to refractory inflammation in UC	Wang et al., Nature, 2020 (59); Flood et al., 2022 (47)
Primary Sjögren’s syndrome (pSS)	IFI16	Significantly upregulated (labial salivary gland biopsies and PBMCs)	Promotes inflammasome (ASC–caspase-1) assembly, inducing GSDMD-mediated pyroptosis and increased IL-1β secretion	Positively correlated with focus score and severity of xerostomia/xerophthalmia; concomitant upregulation of pyroptosis-related genes (GSDMD, CASP1)	Dai et al., Mediators Inflamm, 2025 (46)
Primary Sjögren’s syndrome (pSS)	ZBP1	Increased expression in PBMCs (requires further validation)	Potential activation of necroptosis and NF-κB signaling, enhancing proinflammatory cytokine production (TNF-α, IL-6)	STING pathway exhibits heightened responsiveness in pSS monocytes; ZBP1 may act as a cooperative sensor contributing to excessive inflammation	Huijser et al., Rheumatology, 2022 (61)
Aicardi-Goutières syndrome (AGS)	ZBP1	Pathological activation (central nervous system, systemic tissues)	Loss of ADAR1-mediated RNA editing leads to accumulation of self Z-RNA, triggering ZBP1-dependent PANoptosis and type I interferonopathy	Causative role in severe pediatric neuroinflammation and systemic autoinflammation; ADAR1 mutations are genetically linked to AGS	Jiao et al., Nature, 2022 (34)

## ZBP1 and IFI16 as novel targets for infection therapy

Developing small-molecule agonists to enhance the activity of ZBP1/IFI16 pathway is one promising way for them to be used as vaccine adjuvants or as treatments for the chronic infections in immunocompromised people (8). By activating the cell death dependent on ZBP1 and inflammatory signaling pathways, the ability of the host to clear pathogens may be increased, and this is similar to strategies that improve antitumor immune responses by activating innate immune sensors, like Toll-like receptors (TLRs) (63). However, excessive or uncontrolled inflammatory responses during an infection can easily lead to serious tissue damage and immunopathology. For this reason, developing specific inhibitors to regulate the ZBP1/IFI16 pathways is critical. Small-molecule inhibitors targeting the RHIM domain of ZBP1 or HIN/Pyrin domain of IFI16 can effectively reduce the pathological damage linked to excessive inflammatory responses. They do this by blocking ZBP1/IFI16 interactions with downstream signaling molecules, like RIPK3 (53, 64). This approach aims to keep a balance between the immune defense of host and inflammatory damage, providing new insights for the treatment of severe infections (8, 63).

In murine models of systemic inflammation, the RIPK3 inhibitor GSK’872 can downmodulate necroptosis mediated by ZBP1, and the RIPK1 inhibitor Necrostatin-1 (Nec-1) gives protection against the cell death driven by ZBP1 in intestinal epithelial cells (35, 64). For IFI16, it is reported that the small molecule C16 can stop IFI16 oligomerization and the downstream activation of the inflammasome *in vitro (*53). At the early stage of development, nucleic acid aptamers which target the IFI16 HIN domains have been proposed as competitive antagonists of the binding of dsDNA (28). In preclinical cancer models, researchers explored CRISPR-based ways to delete ZBP1 conditionally (65). To improve the targeting to specific tissues, nanoparticle-based delivery systems (e.g., lipid nanoparticles encapsulating siRNA against ZBP1 or IFI16) have also been explored (66, 67). Cell-type-specific promoters are also used, such as the CD68 promoter for macrophage-restricted expression of dominant-negative IFI16 variants, and PROTAC-based approaches can achieve tissue-selective protein degradation (66–68). Development of reversible and short lived inhibitors may help maintain basic immune surveillance and suppress pathological acute overactivation, offering a route toward clinical translation. Given the dual role of these sensors in protective immunity and pathology, the therapeutic windows may be narrow, requiring precise dosing and timing.

### Applications in autoimmune diseases and cancer

ZBP1 and IFI16 show promise to be used in the fields of autoimmune diseases and cancer immunology. For example, IFI16 can recognize self-DNA, so its dysregulation is linked to how autoimmune diseases (like SLE) start and develop. IFI16 can inappropriately sense self-nucleic acids and activate excessive type I interferon responses and inflammation, leading to tissue damage. Therefore, specific inhibitors targeting the IFI16 pathway could act as workable therapeutic options for these autoimmune conditions. These agents could block IFI16 DNA-binding capabilities or downstream signaling pathways to limit abnormal autoimmune responses (61). In this respect, one important safety consideration is that long term IFI16 inhibition may increase the risk of latent viral reactivation, especially herpesviruses, and also bacterial infections at mucosal surfaces. ZBP1 inhibition for interferonopathies such as AGS is similar, because it may weaken the host defense against RNA viruses, so infections will need to be carefully monitored in clinical settings.

Compared with autoimmune diseases, activating the ZBP1/IFI16 pathway in neoplastic cells may be a promising strategy against cancer in tumor immunology. As ZBP1 can sense Z-nucleotides generated by endogenous retrotransposons and trigger immunogenic cell death characterized by PANoptosis, it favors the release of damage-associated molecular patterns and activates dendritic cells, which promotes the cross-presentation of tumor antigens, so that the anti-tumor immune response becomes stronger (17, 65, 66, 69). This mechanism resembles that of immunotherapies like oncolytic peptides, and it has the potential to reshape the tumor microenvironment (70). Because of this, combining ZBP1/IFI16 agonists with immune checkpoint inhibitors, like anti-PD-1/CTLA-4 antibodies may strengthen antitumor immunity and counteract resistance to immunotherapy (63, 71). However, applications in cancer immunotherapy face several hurdles, such as (1) heterogeneous sensor expression in tumor cells; (2) off-target inflammation in healthy tissues; and (3) autoimmune-like adverse events when combined with immune checkpoint inhibitors. Further preclinical work is needed to validate tumor-specific delivery strategies, such as nanoparticle-encapsulated agonists to safely target ZBP1/IFI16.

### Modulating post-translational modifications of ZBP1 and IFI16

The post-translational modifications (PTMs) for ZBP1 and IFI16 include ubiquitination, phosphorylation, as well as acetylation. They offer fast and reversible ways to control how sensors are activated in infected and stressed cells.

Specific PTM patterns decide whether these sensors drive protective immunity or pathological inflammation, pointing towards new therapeutic strategies. For example, Improving the deubiquitinase USP12 activity could boost antiviral responses mediated by IFI16, while inhibiting ZBP1 deubiquitination might restrain excessive PANoptosis in autoinflammatory disease. The full mapping of PTM landscape of ZBP1 and IFI16 remains a top priority in the field requiring quantitative mass spectrometry across different infection models.

Acetylation of lysine residues within the HIN domains of IFI16 may change its DNA-binding affinity thereby adjusting the threshold for the activation of inflammasome (11, 27). SUMOylation of ZBP1 regulates its nucleocytoplasmic shuttling, but the functional consequences of this in infection models are not fully characterized yet. During apoptosis and pyroptosis, caspase-mediated cleavage of both ZBP1 and IFI16 produces fragments with distinct functions, and these fragments can either stop or redirect the signaling (30, 44).

In respect to phosphorylation, when TBK1 is recruited to IFI16-STING signaling complexes, it phosphorylates IRF3 and IFI16. Phosphorylation of IFI16 at specific serine residues was found to strengthen its interaction with STING, and downstream signal transduction (11, 26). RIPK3 phosphorylates the RHIM domain of ZBP1, which is thought to stabilize ZBP1-RIPK3 complexes, and recruit caspase-8 and MLKL in the process of necroptosis and PANoptosis (21, 31). At the same time, TBK1-dependent phosphorylation of ZBP1 can change its binding preference for Z-RNA or Z-DNA, which confers ligand selectivity under different stress conditions (37).

Further, both ZBP1 and IFI16 undergo regulatory ubiquitination. This influences protein stability, subcellular localization and signaling activity. The deubiquitinase USP12 removes K48-linked polyubiquitin chains from IFI16 to stabilize the protein and strengthen STING-dependent type I interferon responses during viral infection (43). In contrast, the E3 ubiquitin ligase TRIM21 supports the K48-linked ubiquitination and proteasomal degradation of IFI16, in a negative feedback loop to limit sustained immune activation. ZBP1 ubiquitination regulates PANoptosome. For example, in liver cancer models, the E3 ligase KLX mediates ZBP1 ubiquitination, which stabilizes the protein and promotes PANoptosis-mediated tumor suppression (65). The linear ubiquitination of ZBP1-RIPK3 complex by LUBAC regulates how NF-κB is activated downstream of Z-nucleic acid sensing (23).

## Challenges and future perspectives

A major challenge in the field is represented by the lack of systematic knowledge around specific roles of ZBP1 and IFI16 as well as their interactions across different cell types, including immune, epithelial and tumor cells. For example, ZBP1 is shown to promote osteogenic differentiation, while it can inhibit adipogenic differentiation, which shows that its functions depend on the type of cell (72). Another limitation derives from the fact that ZBP1 plays an important role under non-infectious stress conditions like heatstroke and at different stages of infections (38).

Additionally, detecting Z-RNAs still suffers from technical limitations. In this respect the Zα-mFc probe developed recently provides an excellent resource for studying the activation of ZBP1 (7, 8). Still a full exploration of the ligands recognized by ZBP1 and IFI16, especially in non-infectious pathological situations such as metabolic stress and aging is missing. This includes endogenous ligands, for instance Z-RNA: DNA hybrids generated by retrotransposons, and their pathological implications. Other priority areas include: (1) defining the structural and functional features of ZBP1-IFI16 crosstalk, and establishing whether they build multiprotein sensing complexes under specific infection conditions; (2) mapping IFI16 nucleocytoplasmic shuttling mechanisms and the way viral immune evasion proteins regulate them; (3) validating PANoptosis *in vivo* as one pathophysiologically relevant cell death mode during human infectious and autoimmune diseases; and (4) developing isoform-specific and tissue-targeted modulators to reduce toxicity.

Collectively, ZBP1 and IFI16 form a layered and redundant system of nucleic acid surveillance complementing the canonical PRRs like cGAS, AIM2 and RIG-I. Current evidence clearly points to “two roles” of these molecules in immune surveillance. First, they act as sentinels against pathogen invasion. They activate downstream signaling cascades to restrict viral and bacterial replication and dissemination effectively. Second, excessive or aberrant activation results in tissue damage, excessive cytokine release and the breakdown of immune tolerance (67, 68). For this reason, how to preserve their antimicrobial functions while limiting their pathological effects remains a central challenge for future research.

Mounting evidence shows that the activity of ZBP1 and IFI16 is regulated at multiple levels. These levels include post-translational modifications, subcellular localization and interactions with other proteins. Resolving the interaction networks of ZBP1 with molecules such as RIPK3 and MLKL in specific cell types or analyzing the functional differences between nuclear and cytosolic localization of IFI16, may reveal critical checkpoints with important implications for therapeutic intervention.

Small-molecule inhibitors, nucleic acid aptamers and CRISPR-based gene editing technologies may help fine-control the activation thresholds of ZBP1/IFI16, opening four therapeutic opportunities: antiviral, antibacterial, anti-inflammatory or anti-autoimmune.

Avoiding the indiscriminate blockade of these pathways is another challenge (48): Future strategies should prioritize tissue-specific or conditional regulation. For example, nanocarriers may enable targeted delivery of inhibitors to inflammatory sites; at the same time, a critical risk may derive from herpesvirus reactivation upon IFI16 inhibition and increased susceptibility to influenza after ZBP1 inhibition, which may require prophylactic antiviral coverage in therapeutic settings. Finally, increasing attention should be paid to the roles of these sensors in noninfectious diseases like cancer, metabolic disorders and neurodegenerative diseases, which offer clinical impact of ZBP1/IFI16 modulation.

In the next 10 years, advances in single-cell omics, structural biology and artificial intelligence–assisted drug discovery will likely provide deeper insights into the regulatory networks of ZBP1 and IFI16, helping the translation from bench to bedside. For clinicians and drug developers, finding the optimal balance between “precise modulation” and “complete inhibition” of these targets across different disease contexts will be a key challenge. Through this in future it may be possible to harness this core defense network as a powerful tool for precision medicine.
